# Neurosarcoidosis: Clinical, biological, and MRI presentation of central nervous system disease in a national multicenter cohort

**DOI:** 10.1002/brb3.3443

**Published:** 2024-09-15

**Authors:** Amélie Dos santos, Edouard Courtin, Aurélie Ruet, Pierre Duffau, Guillaume Mathey, Ines Bekkour, Jonathan Ciron, Laure Michel, François Xavier Blanc, Jésus Aguilar, Pascal Lejeune, Guillaume Marc, David Laplaud, Armelle Magot, Mohamed Hamidou, Sandrine Wiertlewski

**Affiliations:** ^1^ Service de Neurologie, CRC‐SEP, CHU Poitiers Poitiers Université POITIERS France; ^2^ Service de Neurologie, Institut du Thorax et du Système Nerveux, CRC‐SEP, CHU Nantes Nantes Université Nantes France; ^3^ Service de Neurologie, CHU Bordeaux Bordeaux Université Bordeaux France; ^4^ Service de Médecine interne, CHU Bordeaux Bordeaux Université Bordeaux France; ^5^ Service de Neurologie, CHU Nancy Nancy Université Nancy France; ^6^ Service de Neurologie, CHU Toulouse Toulouse Université Toulouse France; ^7^ Service de Neurologie, CHU Rennes Rennes Université Rennes France; ^8^ Service de Pneumologie, L'institut du Thorax, CHU Nantes Nantes Université Nantes France; ^9^ Service de Neuroradiologie, CHU Nantes Nantes Université Nantes France; ^10^ CHD La Roche sur Yon Service de Neurologie La Roche sur Yon France; ^11^ CHD de St Nazaire Service de Neurologie St. Nazaire France; ^12^ CR2TI, INSERM U1064 Nantes France; ^13^ CHU Nantes, Service des explorations fonctionnelles neurologiques Nantes Université Nantes France; ^14^ CHU Nantes, Service de Médecine Interne Nantes Université Nantes France

**Keywords:** central nervous system, granulomatous, neurosarcoidosis, sarcoidosis

## Abstract

**Introduction:**

Neurosarcoidosis (NS) is a systemic inflammatory granulomatous disease affecting of patients with sarcoidosis. Its diagnosis is difficult as there is no specific test for it. Because of its rarity, the management of NS has so far only been described in case series and short retrospective cohorts. The objective of this study is description of the clinical, paraclinical presentation and the therapeutic management of central nervous system (CNS) involvement in NS patients in France.

**Methods:**

This multicenter, retrospective, observational study involved patients hospitalized between 2010 and 2019 with a diagnosis of sarcoidosis and CNS involvement.

**Results:**

We included 118 patients (38 with isolated NS, 80 with NS associated with systemic sarcoidosis). NS was the initial presentation in 78% of patients, with cranial nerve involvement (36%), medullary symptoms (23%), and seizures (21%). Twenty‐one percent of the patients had already been diagnosed with systemic sarcoidosis. The most frequent biological abnormality was lymphopenia (62.5%), while angiotensin‐converting enzyme was increased in 21%. Meningitis was present in 45% and hyperproteinorachia in 69.5% of cases. MRI mainly revealed white matter abnormalities and leptomeningeal enhancement (34%). Corticosteroids were the most useful treatment, and immunosuppressive agents were used in steroid‐resistant patients and to limit side effects. Methotrexate, cyclophosphamide, and anti‐TNFα were also used, exhibiting good efficacy.

**Conclusions:**

This cohort contributes to a better understanding of the clinical phenotype and associated imaging and biological abnormalities. Sharing of clinical, biological, and imaging data, as well as the therapeutic responses, of patients with NS helps to better understand and manage this disease that affects a small number of patients per center. A database project could be implemented in the future to enable this.

## Graphical abstract

This multicenter, retrospective, observational study describes the clinical, paraclinical presentation and the therapeutic management of central nervous system involvement (CNS) involvement in 118 patients with neurosarcoidosis with CNS involvement.

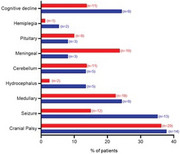

## 1 INTRODUCTION

Sarcoidosis is a systemic inflammatory granulomatous disease characterized by the presence of non‐caseating granulomas (NCG). The annual incidence of sarcoidosis is estimated to be between 1 and 35 per 100,000, and the most frequently involved sites are the lungs and lymph nodes (Prasse et al., 2014). Neurosarcoidosis (NS) accounts for between 5% of sarcoidosis patients based on clinical studies (Prasse et al., 2014) and 50% of patients, based on autopsy studies (Iwai et al., 1993). Isolated NS occurs in 17% of cases (Pawate et al., 2009).

The most useful diagnostic criteria are those of Zajicek (Zajicek, 1999), which are based on the association of clinical, imaging, and pathological results. The diagnosis of NS is particularly difficult because of its polymorphic clinical presentation, the absence of a specific diagnostic test, and the possibility of isolated nervous system involvement (Hoitsma et al., 2004). As a result of the rarity of NS, the clinical presentation and management of this disease have so far only been documented in case series and short retrospective cohort studies (Cohen Aubart et al., 2017; Joseph & Scolding, 2009; Joubert et al., 2017; Leonhard et al., 2016; Nozaki et al., 2012; Zajicek, 1999), (Colover, 1948).

Our objective was to describe the clinical and paraclinical presentation as well as the proposed therapeutic management in cases of central nervous NS from a French, multicenter, retrospective cohort.

## 2 MATERIAL AND METHODS

### 2.1 Population

Patients from six French university hospitals (Nantes, Bordeaux, Rennes, Toulouse, Nancy, and Strasbourg) and two general hospitals (La Roche sur Yon and Saint‐Nazaire) were included in a multicenter, retrospective, observational cohort study.

The inclusion criteria were patients hospitalized between January 1, 2009 and December 31, 2019 with a diagnosis of sarcoidosis with central nervous system (CNS) involvement coded according to the Information Systems Medicalization Program. The codes used were: D86.8: Sarcoidosis of other and associated localization; D86.9: Sarcoidosis; G53.2: Cranial nerve palsy during sarcoidosis. The patients had to fulfill the Zajicek criteria (Zajicek, 1999) for “definite” or “probable” NS.

The exclusion criteria were: patients classified as having “possible NS” according to the Zajicek criteria, patients with another defined neurological diagnosis, and patients with another defined granulomatous disease.

### 2.2 Data collected

A data collection table was created, and we reviewed each medical record. Demographic and clinical data (neurological and systemic symptoms, interval between NS diagnosis and sarcoidosis diagnosis, phenotype of the disease), biological data (blood and CSF), imaging results, and pathology findings were retrospectively extracted from the medical records. The treatment provided and the clinical and radiological responses were also collected.

We constituted two groups: Group 1 with isolated NS at diagnosis and Group 2 with NS associated with systemic sarcoidosis.

### 2.3 Statistical methods

The recorded data are presented as means, medians, and variances for the quantitative variables, and as numbers and percentages for the qualitative variables. For comparisons between groups, we used a non‐parametric Mann–Whitney or a Wilcoxon test for the qualitative variables using GraphPad Prism^®^ (Version 8.0.2) software. A *p*‐value < .05 was considered statistically significant.

### 2.4 Ethics statement

The study was performed under the ethical guidelines issued by the relevant local ethics committees of the participating centers, and the patients provided informed consent for the collection of their clinical information.

## 3 RESULTS

### 3.1 Clinical and demographic characteristics of the patients

**FIGURE 1:**
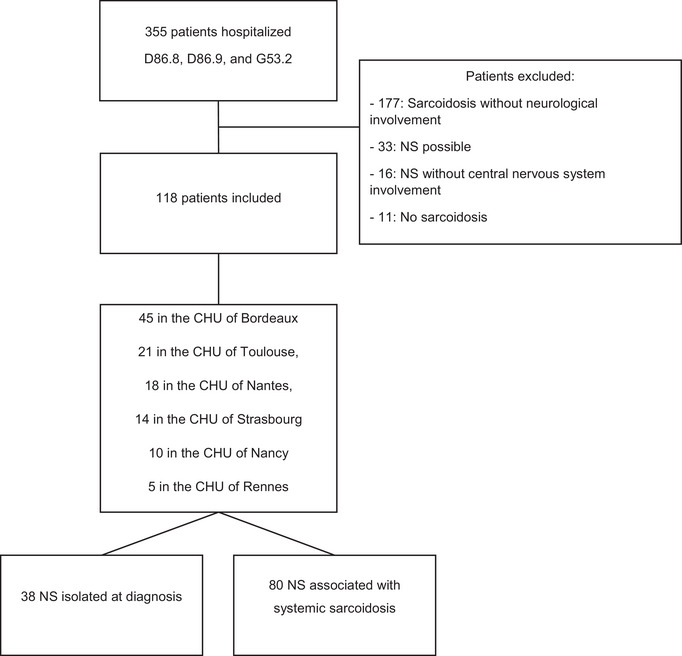
Flowchart. D86.8, Sarcoidosis of other and associated localization; D86.9, Sarcoidosis; G53.2, Cranial nerve palsy during sarcoidosis.

We identified 355 patients and excluded 237 patients after analysis of their medical files (absence of neurological damage, “possible” NS, NS without involvement of the CNS, or absence of sarcoidosis diagnosis). Thus, we analyzed data from 118 patients: 38 in Group 1 (“Isolated NS”) and 80 in Group 2 (“NS associated with systemic disease”) (Table 1). Overall, 26 patients were classified as “Definite” NS and 92 as “Probable” NS. Of the patients with isolated neurological involvement, 20 (52.6%) had “Definite” NS (Figure 1).

**TABLE 1:** Demographic and clinical characteristics.

	Total	Group 1 : Isolated NS	Group 2 : NS associated with systemic sarcoidosis	*p*‐Value
Number of patients Defined NS	118 26	38 20	80 6	
Mean age of NS onset [min–max]	47.1 [8.1‐86.1]	48.1 [8.1−86.1]	46.5 [22−85.6]	.42
Women (%)	47.4%	47.4%	47.5%	.33
Autoimmune comorbidities	12.7%	10.5%	13.7%	>.99
		Hypothyroidism (2) Psoriasis (1) ITP (1)	Hypothyroidism (2) Psoriasis (1) Type 1 diabetes (3) Ankylosing spondylitis (2) Kawasaki disease (1) ITP (1) Autoimmune glomerulonephritis (1) Autoimmune hepatitis (1) Raynaud's syndrome (2)	
First‐degree familial sarcoidosis	1.7%	2.7%	1.2%	>.99
Mean delay in months between first symptoms and NS diagnosis (min–max)	16.7 [0−247]	33.1 [0−240]	9.2 [0−247]	.012*

Abbreviations: NS, neurosarcoidosis; ITP, immune thrombocytopenic purpura.

Mann–Whitney test for age, sex, diagnosis delay comparison. Wilcoxon test for ethnics. * *p* < .05.

The clinical and demographic features were similar between the groups, except for the diagnosis delay between neurological signs and the diagnosis of NS, which was statistically longer in Group 1 (*p* = .012).

### 3.2 Neurological clinical presentation

**FIGURE 2:**
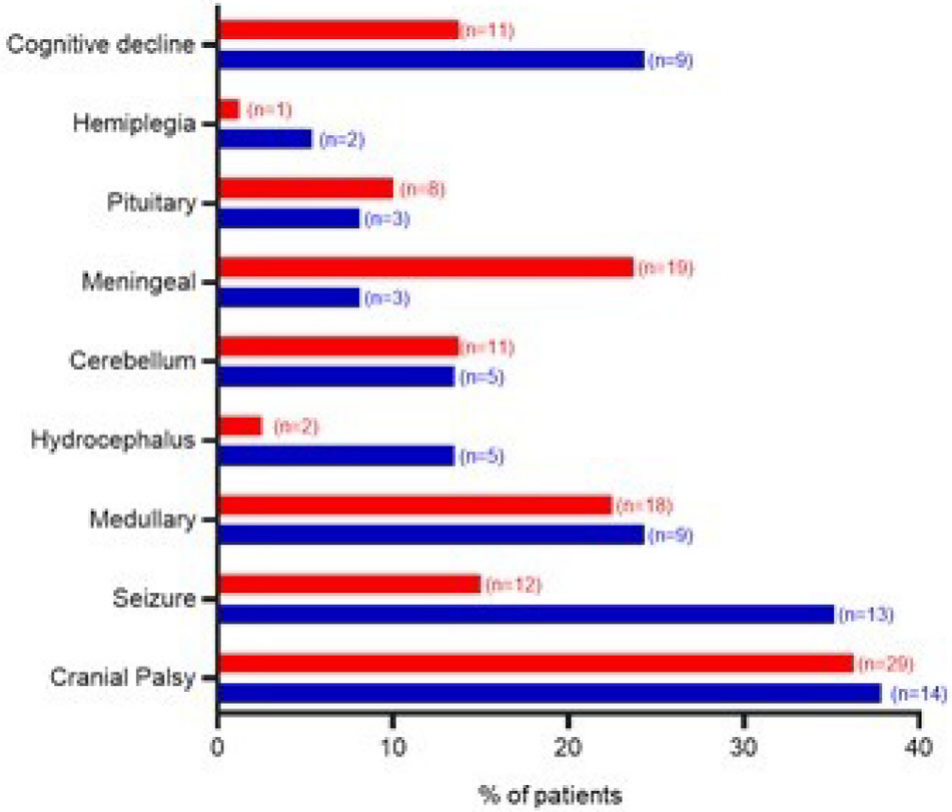
Neurological clinical presentation. Percentage (%) of patients by clinical presentations. In blue: Group 1 (isolated NS); in red: Group 2 (neurosarcoidosis [NS] associated with systemic sarcoidosis). *n*, number of patients.

Neurological involvement was the initial presentation in 77.9% of patients and was isolated in 38 patients (Figure 2).

The most common presentation of NS was cranial nerve palsies in the two groups (36.4%). The facial nerve was involved the most (34.7%), followed by the VIIIth (10.2%) and the optic nerve (6.9%). Multiple cranial nerve neuritis was detected in 39.5% of patients. The second most common presentation was myelitis (22.8%), followed by seizures (21.2%).

**FIGURE 3:**
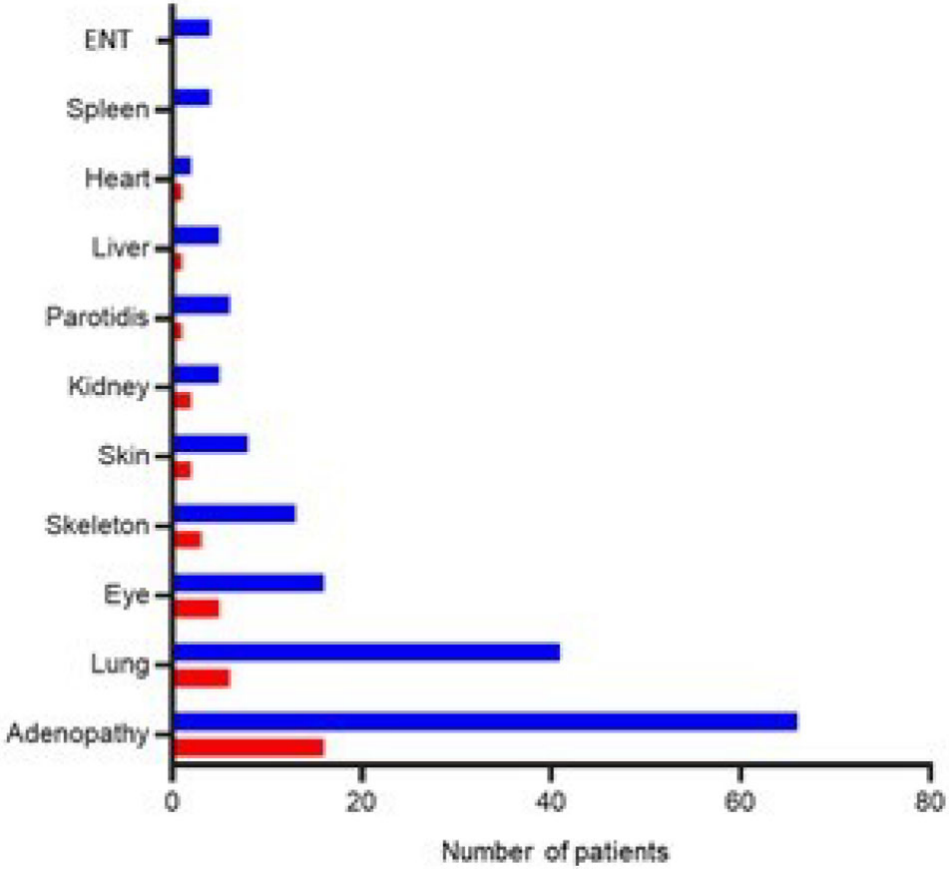
Systemic sarcoidosis associated with neurosarcoidosis. In blue: Group 1 (isolated NS); in red: Group 2 (neurosarcoidosis [NS] associated with systemic sarcoidosis).

In Group 1, 52.8% of the patients were subsequently diagnosed with systemic sarcoidosis (Figure 3), after a mean time of 74.8 months (11−277). Lymphadenopathy was the most common sign (43.2%), followed by lung involvement (16.2%) and ophthalmologic features (13.5%). In Group 2, the diagnosis of systemic sarcoidosis preceded the NS in 20.6% (mean time 151 months [4−1074]) and was concomitant in 48.2% of patients. The most frequent involvements were lymph nodes (mediastinal lymphadenopathy, 70.3%) and lungs (40.7%). Cardiac involvement was diagnosed in just three patients. 

### 3.3 Paraclinical investigations

#### 3.3.1 Biological investigations

The most frequent biological abnormality in our cohort was lymphopenia (62.5%), which was more frequent in Group 2 (*p* = .037). Hypercalcemia and increased ACE occurred in similar proportions in the two groups (Table 2).

**TABLE 2:** Biological results.

		Total	Group 1 : Isolated NS	Group 2 : NS associated with systemic sarcoidosis	*p*‐Value
Blood	Lymphopenia	40/64 (62.5%)	11/26 (42.3%)	28/38 (75,7%)	.037*
	Hypercalcemia	14/90 (15.6%)	4/31 (12.9%)	9/58 (15.5%)	.71
	CRP	36/87 (41.4%)	10/29 (34.5%)	25/57 (43.8%)	.81
	ACE	23/111 (20.7%)	5/36 (13.9%)	18/74 (24.3%)	.055
CSF	Elevated WBC count	42/94 (44.6%)	12/27 (44.4%)	30/67 (44.7%)	.64
	Hypoglycorrhachia	8/64 (12.5%)	1/15 (6.7%)	7/49 (14.3%)	.084
	Hyperproteinorachia	63/97 (64.9%)	18/28 (64.2%)	45/69 (65.2%)	.43
	Oligoclonal bands	21/73 (28,7%)	5/19 (26.3%)	16/54 (29.6%)	.80
	ACEc	13/16 (81.2%)	4/6 (66.7%)	9/10 (90%)	.82

Abbreviations: NS, neurosarcoidosis; CSF, cerebrospinal fluid; CRP, C‐reactive protein; ACE, Angiotensin‐converting enzyme; WBC, white blood cell.

Lymphopenia: lymphocytes count < 1000; hypercalcemia: serum calcium > 2.6 mmol/L; CRP: CRP > 5 mg/L; ACE: ACE > 70 U/L; elevated WBC count: WBC > 5; hyperproteinorachia: proteinorachia > 0.5 g/L; ACEc: CSF ACE > 1 nmol/L.

Mann–Whitney test * p < 0.05.

CSF analysis showed lymphocytic meningitis in 44.6% of cases, hyperproteinorachia in 69.5%, and more rarely hypoglycorrhachia (eight patients in this cohort, including just one case among the patients with isolated neurological damage). Intrathecal synthesis of immunoglobulin was noted in 28.7% of patients and the ACE activity (assayed in 16 patients) was increased in 13 patients.

#### 3.3.2 Biopsy confirmation diagnosis

The patients were biopsied in different organs. The accessory salivary glands were biopsied the most but were also the least informative site (13.1% positivity—69 patients). Biopsy of the mediastinal lymphadenopathy revealed NCG in 89.5% of cases (67 patients). CNS biopsy revealed NCG in 26 (68.4%) of the 38 patients in Group 1.

#### 3.3.3 Brain and spinal MRI

Brain MRI was performed in 113 patients and spine MRI in 65 patients. The results are presented in Table 3.

**TABLE 3:** MRI abnormalities.

	Total	Group 1 : Isolated NS	Group 2 : NS associated with systemic sarcoidosis	*p*‐Value
Brain MRI	***n *= 113**	***n* = 35**	***n* = 78**	
Supra‐tentorial lesions	74 (65.5%)	21 (60%)	52 (66.7%)	.52
Pseudo‐tumoral lesions	13 (11.5%)	7 (20%)	6 (7.7%)	.11
Infra‐tentorial lesions	31 (9.7%)	10 (11.4%)	19 (7.7%)	.73
Lesions enhancement	50 (44.2%)	15 (42.9%)	34 (43.6%)	>.99
Cranial nerve involvement	23 (20.3%)	5 (14.3%)	18 (23.1%)	.32
Pituitary–hypothalamic axis	11 (9.7%)	1 (2.9%)	9 (11.5%)	.16
Hydrocephalus	9 (7.9%)	4 (11.4%)	5 (6.4%)	.45
Meningeal enhancement	38 (33.6%)	12 (34.3%)	25 (32.1%)	>.99
Normal MRI	12 (10.4%)	6 (17.14)	6 (7.7%)	.33
Spine MRI	*n *= 65	*n* = 16	*n* = 49	
Myelitis	27 (41.5%)	8 (50%)	19 (38.8%)	.56
Cervical Thoracic	14 (51.8%) 9 (33.3%)	6 (37.5%) 2 (12.5%)	8 (42.2%) 7 (36.8%)	.08 > .99
Extensive lesions	12 (44.4%)	6 (75%)	6 (31.6%)	.03*
Lesion enhancement	24 (88.8%)	8 (100%)	16 (84.2%)	.24
Meningeal enhancement	10 (15.4%)	4 (25%)	6 (12.2%)	.24
Cauda equine infiltration	5 (7.69%)	1 (6.2%)	4 (8.2%)	>.99
Normal MRI	32 (49.23%)	8 (50%)	23 (46.9%)	>.99

Abbreviations: MRI, magnetic resonance imaging; NS, neurosarcoidosis.

Mann–Whitney test * < 0.05.

#### 3.3.4 Diagnosis of systemic sarcoidosis

Positron emission tomography (PET18F‐FDG) and chest and abdominal tomodensitometry (CT) were performed in 51 and 98 patients, respectively. The former revealed abnormalities in 78.5% of cases: mediastinal diaphragmatic lymphadenopathy (66.3%); hilar lymphadenopathy (31.63%); sub‐diaphragmatic lymphadenopathy (11.2%); pulmonary parenchymal involvement (37.8%); and abnormalities suggestive of the diagnosis at the renal (4/98), splenic (4/98), and hepatic (4/98) levels.

TEP18F‐FDG revealed mediastinal lymph node involvement in 27.5%, hilar in 6.1%, and subdiaphragmatic in 11.2%. The second most frequent systemic involvement was lung involvement in 11.2%. PET also helped to reveal kidney and rheumatological damage.

Forty‐four patients had both examinations, with a better performance of TEP18F‐FDG by highlighting elements not visualized by CT scan.

### 3.4 Treatment

#### 3.4.1 Corticosteroid therapy

The most widely used treatment was corticosteroids (75.42%), at a daily dose of 1 mg/kg. Forty‐one patients were also initially treated with corticosteroid bolus at doses varying between 250 and 1000 mg for 3 days, followed by oral corticosteroids for a mean duration of 21 months (1−132). Complete (16.3%) or partial (34.8%) improvement of symptoms was achieved, with a better effect in the patients of Group 2 (46.4%, *p* = .20).

A new clinical neurological flare was observed in 16 patients during corticosteroid treatment, and 4 patients exhibited progressive worsening of symptoms despite steroid treatment.

Encephalic and/or medullary follow‐up MRI was performed in 52 patients, after a mean duration of 1.13 years following the initiation of corticosteroid treatment. The MRI showed regression of white matter hypersignals in 28.8% of cases and regression of contrast enhancement in 44.2% of the 50 patients who exhibited one initially. The same was observed for 17.3% of the patients exhibiting leptomeningeal contrast enhancement.

New white matter lesions appeared in 14 patients (medullary [3], supratentorial [15.4%], and leptomeningeal [7.7%]), with contrast enhancement in 11.

#### 3.4.2 Non‐steroidal immunosuppressive therapy

Eight patients were treated with an association of corticosteroids and a concomitant first‐line non‐steroidal immunosuppressive therapy (three methotrexate, two mycophenolate mofetil, two cyclophosphamide, and one anti‐TNFα) (Tables 4 and 5). Corticosteroids were initiated as a second‐line therapy with cyclophosphamide in two patients and mycophenolate mofetil in one. Seventy‐nine patients were started with second‐line non‐steroidal immunosuppressive treatment (lack of efficacy of corticosteroids alone), associated with corticosteroid therapy in 81.5% of this subgroup.

**TABLE 4:** Immunosuppressive treatment.

	Methotrexate	Cyclophosphamide	Infliximab	Mycophenolate mofetil	Azathioprine	Adalimumab	Rituximab	Mycophenolic acid
**Number of treated patients**	38	31	27	19	9	9	1	1
**First‐line**	10	13	3	3	0	1	0	0
**2^nd^‐line**	26	14	9	6	5	2	1	0
**3^rd^‐line**	1	4	10	7	3	1	0	0
**4^th^‐line**	1	1	5	2	0	3	0	1
**5^th^‐line**	0	0	0	1	1	2	0	0
**Duration**	3.3 [0.1−18.6]	0.70 [0.08−2.25]	1.19 [0−5.25]	2.7 [0−14.3]	3.2 [0.08−8]	2.17 [0.9−5.9]	NR	NR
**Steroid association**	81.5%	53.4%	66.6%	89.4%	66.6%	33.3%	NR	NR
**Initial dose**	39.6 [0−80]	51.6 [9−80]	30.16 [0−60]	30.4 [5−70]	21 [0−60]	NR	NR	NR
**Final dose**	19.9 [0−80]	17.8 [0−70]	11.6 [0−45]	16.8 [0−60]	5 [0−21]	NR	NR	NR

Number of patients; therapeutic line of treatment: number of patients; duration of treatment in months, with minimum and maximum; Percentage of patients who had a steroid association; initial dose in milligrams; final dose at the end of follow‐up in milligrams.

Abbreviation: NR, non‐related.

**TABLE 5:** Immunosuppressive treatment responses.

	Steroids	Methotrexate	Anti‐TNFα	Cyclophosphamide	Mycophenolate mofetil	Azathioprine	Rituximab
Number of patients	86	38	36	31	20	9	1
Clinical response							
Remission	16.3%	15.8%	8.3%	16.1%	10.5%	0%	0
Improvement	34.8%	28.9%	47.2%	48.4%	52.6%	22.2%	0
Stabilization	23.3%	44.7%	33.3%	25.8%	36.8%	44.4%	0
Worse**n**ing	4.6%	5.3%	2.7%	9.7%	5.3%	11.1%	0
Relapse	22.1%	13.1%	5.5%	6.4%	31.6%	22.2%	0
Reason to stop treatment	57 patients	27 patients	12 patients	31 patients	12 patients	7 patients	NR
Remission	22.8%	18.5%	0%	16%	25%	29.6%	
Stabilization	35.1%	37%	50%	48%	8.3%	42.9%	
Ineffectiveness	38.6%	25.9%	8.3%	24%	33.3%	14.3%	
Adverse event	3.5%	18.5%	41.7%	12%	33.3%	0%	
Adverse events	Weight gain (10.4%) Cushing's syndrome (4.6%) Osteoporosis (4.6%) Psychiatric symptoms (3.4%) Diabetes (1.1%) Pancreatitis (1.1%)	Infections (10.5%) Cancer (5.2%) Death (5.2%) Nausea (2.6%) Weight gain (2.6%) Hepatic cytolysis (2.6%)	Death (8.3%) Infection (5.5%) Myalgia (2.7%) Neoplasia (2.7%) Hepatic cytolysis (2.7%)	Nausea (9,6%) Lymphopenia (6.4%) Cancer (3.2%) Infections (3.2%)	Diarrhea (15%) Hepatic cytolysis (10%) Cancer (5%)	Infections (11.1%)	NR

Number of patients with the treatment during follow‐up; number of patients treated by the line of treatment; duration of treatment: mean [minimum, maximum]; clinical response: % of patients; number of patients who stopped treatment and cause of termination; adverse events, with the percentage of patients.

Abbreviation: NR, non‐related.

##### Methotrexate

Thirty‐eight patients received methotrexate (MTX), including 10 patients in combination with corticosteroids, 26 as a second‐line (after corticosteroid), and 1 patient as a third‐ and fourth‐line therapeutic.

There was an overall good response to treatment, with improvement or recovery in 44.7% of the treated patients, 50% of the 8 patients treated in Group 1, and 52% of the 25 patients in Group 2. Complete clinical recovery occurred in 15.8% of patients.

MRI was performed in 29 patients, with regression of white matter hypersignals and contrast enhancement in 5 patients. Leptomeningeal involvement regressed in three patients. New lesions appeared in seven patients, five of whom had a contrast enhancement.

##### TNFα inhibitors

TNFα inhibitors were used in 36 patients. Infliximab (27 patients) improved symptoms in 55.5% of patients (66% in Group 1 and 47% in Group 2) and resulted in complete recovery in one patient. A control MRI was performed in 18 patients, showing regression of white matter hypersignals in 10 patients and leptomeningeal contrast enhancement in 9 patients. New lesions were observed in seven patients, including two cases of myelitis.

Adalimumab (nine patients) improved symptoms in four patients (44.4%, 0% in Group 1, and 47% in Group 2) and was associated with complete regression in two patients. MRI during treatment was performed in five patients, showing regression of hypersignals in 40% of patients, regression of lesion contrast enhancement in 60%, and leptomeningeal involvement in one patient. New supratentorial lesions were detected in two patients.

##### Non‐conventional immunosuppressive therapies

Based on the data we collected, 25 patients received only corticosteroid therapy, after a mean follow‐up of 36.2 months (3−132). Eight patients did not receive any steroid treatment or non‐steroidal immunosuppressant, five of whom were followed for a mean time of 53.2 months (0−258.5), and three were lost to follow‐up.

#### 3.4.3 Overall progression of the cohort

The average follow‐up time for our cohort was 65 months (0−315). Neurological sequelae were reported in 55.5% of patients, without a difference between the two groups. Eight patients (6%) died during the follow‐up: two from sepsis while taking methotrexate and infliximab, one from suicide, and five from unknown causes.

## 4 DISCUSSION

Our cohort is one of the most substantial published to date for NS, with all patients being diagnosed with anatomical pathological proof (patients with “definite” or “probable” criteria of Zajicek) (Cohen Aubart et al., 2017; Colover, 1948; Ibitoye et al., 2017; Joubert et al., 2017; Nozaki et al., 2012; Zajicek, 1999).

The demographic data and the neurologic presentation (cranial nerve palsies and myelitis) did not differ from the previously described data in the literature nor did they differ between the two groups.

The cognitive presentation is rare and can affect all cognition spheres. No cohort with this presentation has been published to date with systematic neuropsychological testing due, presumably, to the rarity of the pattern. Some cases of young patients exhibiting acute cognitive failure mimicking frontotemporal dementia and confusion have been reported. Moreover, 1.5% with acute dementia, not associated with prion disease, could be associated with sarcoidosis. In our cohort, 16,9% (24.3% in Group 1 and 13.8% in Group 2, without a significant difference) exhibited cognitive failure, with psychomotor slowing and memory failure. Isolated NS occurred in 9 out of 11 cases, making the diagnosis difficult. However, five patients had plurifocal involvement (two medullary involvement, one hemiplegia, and two cranial palsies), leading to a search for a diagnosis other than a degenerative cognitive disorder. Six patients had hallucinations, suggesting a psychiatric pattern. Cranial imaging is paramount (international psychiatric recommendations) but CT is insufficient and can induce a diagnosis trap.

A major challenge of sarcoidosis and NS is the need for biological biomarkers, for diagnosis and also for monitoring the disease. Our results confirm that lymphopenia is most frequently present in patients with systemic involvement and that other biological markers such as serum calcium, CRP, ACE, and the lumbar puncture analysis are non‐specific.

Other biological biomarkers appear to be of relevance in NS: (i) Blood assay of interleukin 2 (IL‐2) receptor, which reflects activation of T lymphocytes. A significant increase in IL‐2 receptor levels in the CSF has been described in NS patients compared with other inflammatory pathologies and in control patients. The level may also be increased in patients with infectious diseases, in particular neuromeningeal tuberculosis, which remains an important differential diagnosis of this disease. An IL‐2 receptor level greater than 150 pg/mL is associated with a sensitivity of 61% and a specificity of 93% for the diagnosis of NS (Petereit et al., 2010); (ii) assay of the CSF CD4/CD8 ratio, with parallel use of this assay in pulmonary sarcoidosis diagnosis. There is a significant increase in the CD4/CD8 ratio in the CSF of patients with NS compared to other inflammatory diseases of the CNS: a ratio >3.9 is associated with a sensitivity of 28.57% and a specificity of 87.2% for the diagnosis of NS (Chazal et al., 2019).

Concerning MRI, non‐specific T2 lesions (periventricular locations, central gray nuclei, cortical or juxta‐cortical) are the most frequent abnormalities reported in the literature (74%−83%) (Fritz et al., 2016). Enhancement (nodular, with a complete or incomplete ring) was present in 44.2% of cases in our cohort. Indeed, meningeal, leptomeningeal, and pachymeningeal enhancement were frequently found on T1 sequences with an injection of gadolinium, particularly at the skull base. This was seen in 33.6% of our patients, and it represented the only abnormality on MRI in 10 patients. We were surprised that patients from this cohort did not receive systematic follow‐up MRIs. We believe that it would be useful for patients to undergo a control MRI at least one year after the initial diagnosis to document the regression of lesions.

Spectrometry can differentiate the granulomatous lesions of sarcoidosis from infectious lesions such as tuberculosis (Sammarra et al., 2019). Indeed, a decrease has been reported in the N‐acetyl‐aspartate/creatine ratio without an abnormal peak, unlike tumor lesions, for which there was a collapse of these peaks and an increase in the choline and lactate peaks. In terms of infectious lesions, there was an increase in lactate, lipids, and amino acids.

Neurological impairment secondary to sarcoidosis remains a difficult proposition. Hoitsma et al. (Hoitsma et al., 2004) discussed three clinical situations:

Systemic sarcoidosis diagnosis before or during neurological involvement with the presence of signs of extra‐neurological activity;

The same situation but with no signs of activity;

The onset of neurological symptoms in a patient without a known diagnosis of systemic sarcoidosis.

The first situation was found in 20.7% of our patients. “Active” systemic sarcoidosis led to a link with neurological signs; “inactive” sarcoidosis requires being very thorough in terms of a possible differential diagnosis. Evidence of systemic impairment is a major issue in patients with initial neurological impairment and suspected sarcoidosis, with 48.3% of concomitant systemic diagnoses.

The most performed sample for identification of gigantocellular epithelial granulomas without caseous necrosis in our cohort was salivary accessory gland biopsy. Positivity was present in only 13.1% of patients. In addition, 29.4% of patients had lymphocytic sialadenitis with a focus score > 1, which could be a guiding element. In our cohort, the diagnostic performance of mediastinal lymphadenopathy biopsies was better than that of salivary gland biopsies, with 89.5% positivity. Where feasible, this biopsy site should, therefore, be selected more frequently.

Therapeutic management of patients with NS is not consensual. In our cohort, there was no difference between the two groups regarding treatment management and therapeutic responses. Corticosteroids were the most widely used treatment, being used in 75.4% of our treated patients. The beneficial effect of corticosteroids is well established, notably in pulmonary sarcoidosis (James et al., 1967; Paramothayan et al., 2005). Corticosteroids have many adverse effects, however, and may be insufficient to achieve remission in patients with NS. It was, therefore, necessary to consider non‐steroidal immunosuppressive treatments. To date, there are no guidelines for the optimal timing or the class of immunosuppressive treatment to use first.

The most commonly used non‐steroidal immunosuppressive treatment in the literature and our cohort was methotrexate. This is the only treatment for which randomized placebo‐controlled studies have been conducted. In our cohort, 15.8% of patients treated with methotrexate were in complete remission, and 28.9% could be considered to be partially improved. A randomized study that compared MTX to mycophenolate mofetil found a lower annualized relapse rate for the group treated with methotrexate (Bitoun et al., 2016).

Cyclophosphamide is a therapeutic alternative to obtain a high percentage of remission. In a small series, Doty et al. (Doty et al., 2003) showed remission in four out of seven patients and improved imaging and lumbar punction in all patients. We also found that cyclophosphamide was a steroid‐sparing agent. However, its use remains limited due to significant adverse effects, impacts on fertility, and the cumulative maximum dose of 24 g.

A number of recent studies have demonstrated the efficacy of anti‐TNFα, including infliximab, in the treatment of NS. TNFα expression can increase in patients with sarcoidosis, thereby promoting the formation and maintenance of granulomas. In 2017, Pawate et al. (Gelfand et al., 2017) conducted a multicenter, retrospective study in 66 patients treated with infliximab (associated with another oral immunosuppressant in 78.8%). This regimen resulted in complete remission at one year in 28.8% of patients and improved symptoms in 48.5% of patients, with improved MRI in 69.7% of cases. When infliximab was stopped, because of clinical remission or adverse effects, recurrence of neurological symptoms occurred in 50% of cases (Fritz et al., 2016; Gelfand et al., 2017). The use of anti‐TNF drugs implies diagnostic certainty, as this type of treatment can exacerbate other inflammatory diseases such as MS. It remains necessary to formally eliminate an infectious granulomatous disease or a demyelinating inflammatory pathology before initiating anti‐TNFα treatment (TNF neutralization in MS: results of a randomized, placebo‐controlled multicenter study, 1999). Adalimumab (Hutto et al., 2022) appears to be effective, but the literature to date has only reported case reports or small series of patients. The phase III therapeutic trial EFIRTES investigating the efficacy of infliximab versus placebo in the treatment of extra‐thoracic disease is currently underway in France (NCT03704610).

Sharing of clinical, biological, and imaging data, as well as the therapeutic response of patients, also appears to be important for gaining a better understanding of this disease, because of its rarity and the small number of patients per center. A national database project could be implemented. The relevance of such a database would be to provide a systematic treatment monitoring plan (the approximate date of MRIs, for example), homogeneous corticosteroid therapy management, alone or in combination with non‐steroidal immunosuppressive treatments as a first‐line and proposals for subsequent “lines” of immunosuppressive treatment.

## AUTHOR CONTRIBUTIONS

Amélie DOS SANTOS and Sandrine WIERTLEWSKI Conceived the design of the study, Collected the data, Contrbuted data or analysis, Performed the analysis, Worte the paper, Discussed the results, Contributed to the final manuscript. Edouard Courtin, Ines Bekkour, Laure Michel, Jonathan Ciron Conceived the design of the study, Collected the data, Discussed the results, Contributed to the final manuscript. Pascal Lejeune and Guillaume Marc Collected the data, Discussed the results, Contributed to the final manuscript. Aurélie Ruet, Pierre Duffau, Guillaume Mathey, Francois Xavier Blanc, Jesus Aguilar, David Laplaud, Armelle Magot and Mohamed Hamidou Discussed the results, Contributed to the final manuscript.

## CONFLICT OF INTEREST STATEMENT

The authors have no financial disclosure to declare.

### PEER REVIEW

The peer review history for this article is available at https://publons.com/publon/10.1002/brb3.3443.

## Data Availability

The data that support the findings of this study are available from the corresponding author, Dr. Dos Santos Amélie, upon reasonable request.
